# Behavioral and psychological impact of returning breast density results to Latinas: study protocol for a randomized clinical trial

**DOI:** 10.1186/s13063-019-3939-6

**Published:** 2019-12-18

**Authors:** Bhavika K. Patel, Jennifer L. Ridgeway, Karthik Ghosh, Deborah J. Rhodes, Bijan Borah, Sarah Jenkins, Vera J. Suman, Aaron Norman, Matt Jewett, Davinder Singh, Celine M. Vachon, Carmen Radecki Breitkopf

**Affiliations:** 1 Mayo Clinic, Phoenix, AZ USA; 2 Mayo Clinic, Rochester, MN USA; 3 Mountain Park Health Center, Phoenix, AZ USA

**Keywords:** Mammographic breast density (MBD), Mammography, Breast density, Breast density legislation, Breast cancer, Hispanic, Randomized clinical trial (RCT)

## Abstract

**Background:**

Breast cancer is the most common cancer and the leading cause of cancer mortality among Latinas. As more is learned about the association between mammographic breast density (MBD) and breast cancer risk, a number of U.S. states adopted legislation and now a federal law mandates written notification of MBD along with mammogram results. These notifications vary in content and readability, though, which may limit their effectiveness and create confusion or concern, especially among women with low health literacy or barriers to screening. The purpose of this study is to determine whether educational enhancement of MBD notification results in increased knowledge, decreased anxiety, and adherence to continued mammography screening among Latina women in a limited-resources setting.

**Methods:**

Latinas LEarning About Density (LLEAD) is a randomized clinical trial (RCT) comparing the impact of three notification approaches on behavioral and psychological outcomes in Latina women. Approximately 2000 Latinas undergoing screening mammography in a safety-net community clinic will be randomized 1:1:1 to mailed notification (usual care); mailed notification plus written educational materials (enhanced); or mailed notification, written educational materials, plus verbal explanation by a *promotora* (interpersonal). The educational materials and verbal explanations are available in Spanish or English. Mechanisms through which written or verbal information influences future screening motivation and behavior will be examined, as well as moderating factors such as depression and worry about breast cancer, which have been linked to diagnostic delays among Latinas. The study includes multiple psychological measures (anxiety, depression, knowledge about MBD, perceived risk of breast cancer, worry, self-efficacy) and behavioral outcomes (continued adherence to mammography). Measurement time points include enrollment, 2–4 weeks post-randomization, and 1 and 2 years post-randomization. Qualitative inquiry related to process and outcomes of the interpersonal arm and cost analysis related to its implementation will be undertaken to understand the intervention’s delivery and transferability.

**Discussion:**

Legislation mandating written MBD notification may have unintended consequences on behavioral and psychological outcomes, particularly among Latinas with limited health literacy and resources. This study has implications for cancer risk communication and will offer evidence on the potential of generalizable educational strategies for delivering information on breast density to Latinas in limited-resource settings.

**Trial registration:**

ClinicalTrials.gov, NCT02910986. Registered on 21 September 2016.

Items from the WHO Trial Registration Data Set can be found in this protocol.

## Background

Breast cancer is the most common cancer and the leading cause of cancer mortality among Hispanic women/Latinas [1]. Efforts to inform Latinas about screening options to improve early detection of breast cancer and educate women who are at higher risk are important and remain understudied, particularly among less acculturated Latinas [2]. Research also shows lower knowledge of mammographic breast density (MBD) among Latinas [3]. Increased MBD is a strong risk factor for breast cancer and is associated with reduced sensitivity of mammograms and delays in diagnosis [4]. Women with dense breasts may benefit from supplemental screening, but presently, there are no clinical guidelines for supplemental screening [5]. A powerful movement led by breast cancer survivors and patient advocates has led to 36 U.S. states adopting state legislation mandating [6] that women be informed of their breast density and dictating the method (mailed letter) and notification language.

Adoption of state legislative notification mandates since 2009—along with a new federal mandate—have progressed while research on the impact of such notification is still emerging [7, 8]. Research is particularly critical for populations known to be vulnerable to health disparities such as racial/ethnic minorities and those with lower health literacy, limited English proficiency, and socioeconomic disadvantage. The effect of providing women with notification about their MBD and its impact on breast cancer risk without accompanying education may have unintended consequences, particularly among Latinas with lower health literacy and limited resources. Two recent qualitative studies with Hispanic women found that MBD notification language was confusing and led to misinterpretation, including misunderstanding of key concepts like masking and breast cancer risk [9, 10]. While MBD notification language elicited worry and anxiety among some women, the majority in one of the studies reported a desire for MBD notification and potential for it to influence future screening, including pursuit of supplemental screening modalities [10]. The likelihood of disparities emerging between women who can pursue supplemental breast screening and women who are less able to do so is also an important consideration [11].

This study examines important outcomes among women of relative socioeconomic disadvantage by implementing a randomized clinical trial (RCT) that compares usual care to two “educationally enhanced” approaches to notification. It includes a comprehensive set of longitudinal outcome measures and qualitative inquiry and cost analysis to enhance our understanding of delivery of an educational intervention in practice and its transferability to other healthcare settings. Conducting this study in the context of newly enacted legislation and in a community setting that provides mammography screening to Latinas who are under-insured or uninsured will provide critical data that can inform future policy at the state and national level and impact clinical practice. This study takes place in a federally qualified health center (FQHC) in the USA. FQHCs are community-based, safety-net health centers that provide primary and preventive care for medically underserved populations regardless of their ability to pay [12]. Obtaining answers to these questions in the context of a community clinic such as Mountain Park Health Center (described below) will provide data that are currently lacking on approaches to optimize density notification for vulnerable populations.

## Methods/design

### Study aims

This is an RCT to assess the impact of three different approaches to breast density notification: mailed notification (usual care) versus (vs.) mailed notification plus written educational materials (enhanced) vs. mailed notification, written educational materials, plus verbal explanation and education by a lay health educator/*promotora* (interpersonal).

1. Specific aim 1: compare anxiety as well as knowledge gained between different breast density notification approaches (usual care (UC), enhanced, interpersonal). We hypothesize that Latinas randomized to the interpersonal group (receiving a density notification letter that is accompanied by written educational materials, plus interaction with a *promotora*) will have less anxiety and more knowledge gained relative to either the UC or enhanced study groups.
2. Specific aim 2: compare adherence to attending the next routine screening mammogram between different breast density notification approaches (UC, enhanced, interpersonal). We hypothesize that Latinas randomized to the interpersonal group (receiving a density notification letter that is accompanied by written educational materials, plus interaction with a *promotora*) will be more likely to adhere to attending the next screening mammogram compared to either the UC or enhanced study groups.
3. Specific aim 3: whether or not the interpersonal group is found to have more favorable outcomes, we will examine the experience of the *promotora* in order to understand their conversations with patients, identify patients’ concerns about their notification, and understand contextual factors related to implementation of the intervention. If the interpersonal approach is found to be successful, this aim will inform refinement of the educational intervention for dissemination. If the interpersonal approach is not found to be successful, this aim will provide insight on potential shortcomings. This aim involves qualitative inquiry.

4. Exploratory aim: estimate the financial impact of the interpersonal *(promotora)* intervention by performing a cost analysis.

### Research setting

The study is being conducted at Mountain Park Health Center (MPHC), the largest FQHC in Phoenix, AZ. MPHC works with the communities it serves, including health and social service agencies, academic institutions, local foundations, and government entities, to assist patients in receiving the medical services they need at a price they can afford. As an FQHC, MPHC accepts Medicaid and Medicare and is able to offer services through a sliding-fee scale. No patient is turned away for inability to pay. Nearly 2000 women per year (85% Hispanic) receive screening mammography at MPHC. Mayo Clinic radiologists read the results of mammograms completed by the MPHC radiology technician, and since 2012, Mayo Clinic and MPHC have collaborated on research projects related to MBD and cancer risk among Latinas.

The State of Arizona adopted breast density notification in 2014 (AZ Rev. Stat 36–415). The notification language states: “Your mammogram indicates that you have dense breast tissue. Dense breast tissue is common and is found in fifty percent of women. However, dense breast tissue can make it more difficult to detect cancers in the breast by mammography and may also be associated with an increased risk of breast cancer. This information is being provided to raise your awareness and to encourage you to discuss with your health care providers your dense breast tissue and other breast cancer risk factors. Together, you and your physician can decide if additional screening options are right for you. A report of your results was sent to your physician.”

### Trial oversight

Trial design and conduct are overseen by the trial steering committee, which consists of the Principal Investigator (chair), Co-Investigator(s), and study statistician. The Principal Investigator and study coordinator prepared documents related to human subject protections and all members of the steering committee reviewed and agreed upon the final protocol, including recruitment and consent procedures and documents, and all subsequent revisions to the protocol. The steering committee meets monthly to review study progress, including recruitment and survey response rates. All changes to study conduct are approved by the Principal Investigator with consultation from the steering committee members. The Principal Investigator and study statistician will hold and maintain the final dataset. Substantive contributions to the design, conduct, interpretation, and reporting will be recognized through the granting of authorship on publications from this trial. There is no plan to use professional writers. Disputes on authorship will be settled by the Principal Investigator after consultation with the steering committee.

### Interventions

#### Usual care

Women in the UC group receive the mammogram results letter that is part of standard practice in this setting. It includes the the aforementioned statement about breast density results.

#### Enhanced intervention (enhanced)

A written educational brochure about breast density was created for this study in both English and Spanish. Informed by the clinical expertise of study team members, the brochure outlines the meaning and implications of dense breast tissue. It includes photos demonstrating the categories radiologists use to describe dense tissue, and it was reviewed by institutional patient education experts for readability. Study personnel mail the brochure along with the mandated mammogram results letter (which includes the aforementioned statement about breast density results).

#### Interpersonal intervention (interpersonal)

The written educational brochure about breast density given to the enhanced group is also given to this group. This group also receives telephonic delivery of breast density education by a trained health educator (i.e., *promotora*). *Promotora* education has been used in a variety of clinical contexts and has been shown to have positive effects on Latinas’ behavior, risk perception, satisfaction with care, and knowledge, including that surrounding breast cancer prevention [13–17]. A recent systematic review of community health worker (CHW/*promotora*) interventions in the mammography context reported that 8 of 11 studies favored CHW intervention vs*.* print or mailed educational materials, with an overall “moderate” strength of evidence [18]. It also stressed the importance of incorporating conceptual models and corresponding measures in future RCTs to enable evaluation of the mechanism through which *promotora* interventions impact outcomes, methodologic rigor (e.g. blinded evaluation, fidelity checking), and evaluation of intervention costs [18]. The *promotora* educational script was informed by clinical experts on the study team, and by the information-motivation-behavioral skills (IMB) model [19], which posits that accurate information and personal motivation influence behavior directly and indirectly by activating behavioral skills/efficacy. The *promotora* contacts women by telephone approximately 2 weeks after randomization (to allow time for results to be available and for women to receive the mailing). During the telephone call, she presents educational information, including a review of information in the mailed brochure, and she answers questions or corrects misinformation as needed. She also delivers motivational messages related to adherence to breast screening and family health.

### Patient recruitment and consent

Women between 40 and 74 years of age, who self-identify as Hispanic/Latina, and who speak English or Spanish, and who are presenting for a screening (vs. diagnostic) mammogram are eligible for this study. Study coordinators perform a review of the electronic health record (EHR) of women scheduled for appointments for eligibility. Women who meet study eligibility are contacted by phone by study staff at least a week prior to their appointment, to ascertain women’s interest in hearing more about the study. Interested women are scheduled to meet with a trained Spanish/English bilingual study coordinator when they arrive at the MPHC mammography unit for their screening mammogram. If the patient is willing to participate, she is asked to provide written informed consent and authorization to release medical records for this research study. The informed consent and authorization document, which is available in English and Spanish, includes study contact information and a description of study procedures, and information about the rights of research participants, the potential risks and benefits of participating in the research, and procedures that will be used to protect privacy and confidentiality. It also outlines how participant health information will be used or shared with others. The Mayo Clinic Institutional Review Board (IRB) approved the informed consent and authorization document. The Principal Investigator completes annual continuing review reports to the IRB that include information about study status, accruals, and withdrawals, and any unanticipated problems or events. The informed consent and authorization document is updated annually with IRB approval.

Any modifications to the study protocol (e.g., eligibility criteria), participant contact materials, or data collection instruments are approved by the steering committee and the IRB, as appropriate, before implementation in the study. Institutional IRB policies require that participants be notified of changes risk, which are not anticipated with the educational nature of the interventions in this study. Adherence to the educational interventions will not be monitored or measured, and concomitant care and post-trial care are not applicable for this study. Handling of biological specimens and plans for future ancillary studies using biological biological samples are not applicable to this trial as no samples will be collected. Future studies using these data outside of the aims of the trial must be submitted to the IRB for approval.

### Randomization

**Fig. 1:**
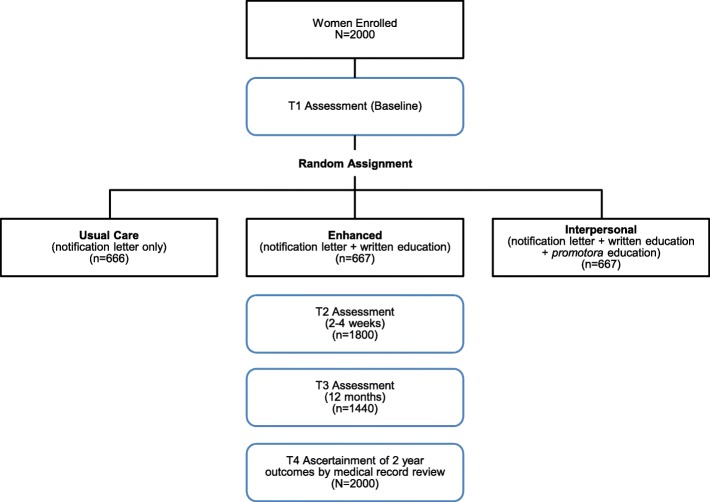
Flow diagram showing study groups, anticipated sample sizes, and assessment time points

Block randomization is used to assign participants to a study arm where the stratification factors are age ≥ 50 years (yes vs. no); ethnicity (Hispanic vs. non-Hispanic), and language preference (Spanish vs. English), with a block size of 6 for each factor combination. Women are randomized after giving consent, using an algorithm within Medidata Rave®, which is a Health Insurance Portability and Accountability Act (HIPAA)-compliant data management system. The randomization scheme is implemented within Medidata Rave using an integrated randomization module (Medidata RTSM), and the random allocation occurs automatically within the system after the consent date is entered. The use of general nomenclature (group 1, 2, 3) is used to maintain blinding. The study assistant administering survey assessments is blinded to study group, although it is possible participants will reveal their group identity to the assistant. The data analysts and statisticians are not blinded to study group, to ensure accurate tracking and data checks during the study. Tracking of patient consent and random assignment and compliance with the study calendar are also managed in Medidata Rave®. The accrual goal is 2000 women (See Fig. 1).

## Methods: aims 1 and 2

There are four post-randomization assessment time points, as shown in Table 1 (see Additional file 1 for the Standard protocol items: recommendation for interventional trials (SPIRIT) checklist). Selection of survey outcome measures was guided by the IMB framework, such that the measures address each of the model constructs (information, motivation, and behavior). More specifically, receiving MBD results in the context of a conversation that delivers information dynamically, corrects misperceptions, provides personal and social motivation to act, and builds self-efficacy for behavioral performance is likely to be more effective than factual information delivered only in writing (enhanced group) or notification only (UC group). Therefore the selected measures are aimed at assessing the mechanisms by which information influences motivation and behavior within the IMB model, (e.g., self-efficacy to return for mammography, intention to adhere to screening recommendations, and intention to discuss MBD with a healthcare provider), as well as potential moderating factors such as depression and worry about breast cancer which, among Latinas, are linked to diagnostic delays in the context of mammography [20, 21].

**Table 1:** Flow of study procedures

	Study period					
	Enrollment	Allocation	Post-allocation			
Timepoint	*-T*_*1*_	0	*T*_*1*_	*T*_*2*_	*T*_*3*_	*T*_*4*_
Enrolment:						
Eligibility screen	X					
Informed consent	X					
Allocation		X				
Interventions:						
Enhanced (letter + brochure)			X			
Interpersonal (letter + brochure + *promotora*)			X			
Assessments^a^:						
Age, ethnicity, language preference (EHR)	X	X				
Mammogram results, clinical characteristics (EHR)			X			
Demographics and personal/family health history			X			
STAI and related psychological measures			X	X	X	
PROMIS short form depression			X	X	X	
MDB knowledge			X	X	X	
Acceptability of MBD results information delivery				X		
Self-reported knowledge of MBD results				X		
Intention to adhere to mammography screening				X	X	
Discussions with healthcare provider				X	X	
Information seeking about MBD					X	
Self-reported mammography adherence					X	
Mammography adherence and follow up (EHR)					X	X

*EHR* electronic health record, *STAI* State-Trait Anxiety Inventory, *PROMIS* Patient-Reported Outcomes Measurement Information System, *MBD* mammographic breast density

^a^Assessments are self-report surveys unless noted as EHR (chart abstraction)

### T1, T2, and T3 survey assessments

T1: the T1 survey is a paper-based assessment administered in person by a bilingual member of the study staff. Based on patient preference (and literacy levels) patients can complete the survey on their own or ask the study coordinator to administer it verbally. Mode of administration is tracked in the study database. The survey includes items on demographic characteristics, family history of breast cancer [22], mammography screening history (approximate lifetime number of mammograms, number of times recalled, number of breast biopsies), and anxiety as measured using the state anxiety subscale of the State-Trait Anxiety Inventory (STAI) [23, 24]. The STAI was selected because of its known psychometric properties, literature on its use in mammography [25, 26], and the study team’s experience with its use in an intervention study involving Latinas attending cancer screening [27] Additional psychosocial measures include health literacy, [28–30] cancer fatalism, [31] self-efficacy to return for mammography at the next screening interval, worry about breast cancer [32, 33], perceived risk of breast cancer using numerical, verbal, and comparative estimates [34], and the Patient-Reported Outcomes Measurement Information System (PROMIS) measure (short form) addressing depression [35]. To understand whether educational strategies are advantageous relative to written MBD notification, we also assess knowledge using three items: one regarding MBD as a risk factor, one related to MBD having a masking effect (both previously used in MBD knowledge assessment) [3], and one novel item regarding mammography screening intervals.

The T1 assessment period also includes data abstraction of patient demographic and clinical characteristics. Data obtained from the EHR at T1 include breast density category, screening mammogram results (normal or abnormal), body mass index, parity, age at menarche, menopausal status, hormonal contraceptive use, breastfeeding, and age at first birth.

T2: the second assessment (T2) for the UC and enhanced groups is a telephone survey administered approximately 2 weeks post-randomization, which corresponds to the expected timeframe for receiving the mailed mammogram and breast density results and educational brochure. For the interpersonal group, the T2 survey is administered after the mailed mammogram and breast density results and educational brochure has been delivered and the *promotora* has completed the telephone education call or exhausted attempts to do so (approximately 4 weeks). The T2 survey is designed to measure acceptability of the method by which women received their MBD results and satisfaction with the content and clarity. Knowledge is re-assessed using the same items used in the T1 assessment. Women are queried about their own breast density as a check that the mailed notification was read and processed. Anxiety, depression, perceived risk, self-efficacy, and measures of worry about breast cancer that were administered at T1 are also repeated. Behavioral intention to return for a screening mammogram at the recommended interval is assessed using items developed by Lerman [32]. All women are asked if they discussed their breast density with their health care provider—a described aim of most notification state laws. Women with dense breasts are asked if they discussed supplemental screening options with their healthcare provider (e.g., breast ultrasound, magnetic resonance imaging (MRI), tomosynthesis, molecular breast imaging (MBI)). This information is especially important to understand in a resource-constrained setting like a FQHC.

T3: 1 year post-randomization all groups undergo the final survey assessment (T3), which is telephone administered. Participants are contacted by the study coordinator approximately 1 year after density notification letters are mailed. The T3 assessment includes repeated measurement of anxiety, depression, worry about breast cancer, perceived risk, and discussion of their MBD with their healthcare provider. It also includes questions on information seeking about the topic of breast density and re-assesses participants’ knowledge on MBD. Intentions on future utilization of mammography and supplemental breast screening are also measured. Attendance at mammography is queried at T3 to capture self-reported screening behavior that may have occurred elsewhere and would not be documented in the MPHC EHR. Self-report of attendance at mammography will be corroborated with EHR data.

### T3 and T4 chart abstraction

Adherence to subsequent mammography screening or follow-up testing (diagnostic mammography, breast ultrasound), and delay in attending follow-up care will be ascertained from the EHR T3 and T4 assessments (1 and 2 years post randomization). As recommended screening intervals differ by major consensus groups and may be annual or biennial [36] patient adherence to the recommended screening interval will be determined based upon review of the clinic note. MPHC followed annual screening guidelines, starting screening at age 40 years at study launch. Further, as delays in diagnosis may contribute to disparities in breast cancer outcomes [20, 37], the number of weeks of delay in attending a follow-up appointment will be recorded as a “time to adherence” variable; women who are adherent will be assigned a delay score of “0.” It has been shown that delays in diagnosis of breast cancer as short as 3 months are associated with decreased survival [37, 38], therefore we will also consider delay of more or less than 3 months as a dichotomous variable (delayed or not delayed). Any uptake in supplemental breast screening among women with dense breasts will also be captured in the EHR review.

## Methods: aim 3

Qualitative approaches are used in RCTs of clinical interventions to gain in-depth understanding of the implementation of the intervention, including contextual factors that promote or inhibit outcomes, providing critical information in interpreting trial results, and advancing knowledge translation [39, 40]. It is also optimal for understanding the complexity and nuances of new interventions in their “natural” context [41] and offering novel insights that might otherwise not be captured or studied [42]. This approach is particularly appropriate for studying the implementation of the *promotora* intervention, which is based on dynamic conversations, as it can shed light on how women perceive MBD and the questions they have related to MBD, cancer risk, and screening options. Our results will add to knowledge of how best to deliver density information to vulnerable populations. Additionally, these data can inform understanding of implementation variation or adaptation, aid in interpretation of the primary study outcomes, and provide contextual information underscoring transferability of findings to other settings.

This study employs a narrative approach that focuses on the experience of the *promotora.* Following each patient interaction, the *promotora* completes documentation in a HIPAA-compliant electronic database (Research Electronic Data Capture (REDCap)) [43]. Each entry documents standard information about the conversation, including structured fields for capturing participant-reported intentions to speak with others about MBD or family history of breast cancer. Open-ended entries allow for more detailed descriptions of patient questions or concerns related to MBD or other topics participants may view as related, such as cancer risk. The *promotora* also uses an electronic diary to document her perceptions of the intervention and its delivery. This process also acts as an electronic study log for capturing any adaptations or variations to the intervention over time. This aim focuses on the *promotora’s* point of view, as the one delivering the intervention. Patient outcomes are identifiable and captured by quantitative measures used in aims 1 and 2. The qualitative data collected for aim 3 will aid in interpreting patient outcomes, such as those related to knowledge or perceptions of risk.

## Methods: exploratory aim

Obtaining cost data is necessary for translating research into practice and estimating sustainability. In order to assess the feasibility of implementing the *promotora* intervention in clinical practice, the primary incremental cost will include the hourly labor cost of training and employing the *promotora*. The average cost of *promotora* time per patient will be estimated using data on call duration that the *promotora* documents in the electronic database. The density notification letter plus written educational materials (“enhanced” study group) is not expected to incur any additional cost, as the educational materials are included with the mammography result and density notification mailing. However, the study team will monitor any unexpected costs associated with this work.

### Statistical considerations

This study is collecting a rich set of data for quantitative analysis surrounding the return of MBD results, including PROs and EHR-documented longitudinal behavioral outcomes. These data include a set of continuous/ordinal responses that measure psychological outcomes (anxiety, worry about breast cancer, perceived risk), and knowledge of MBD as a breast cancer risk factor and acceptability/satisfaction with the method of notification. The data will also include categorical behavioral responses assessing whether each woman adhered to screening mammography at their recommended interval, attended (with or without delay) recall for an abnormal screening mammogram, and discussed MBD with their provider. Participants randomized to the interpersonal arm but unable to be reached for intervention delivery will be allocated to the enhanced arm for analysis.

### Sample size considerations and statistical power

A total of 2000 Latinas will complete enrollment and be randomized to the three study groups (approximately 667 women per group). Applying a 10% attrition rate between enrollment and the first follow-up time point and an additional 20% after 1 year, we have planned for a sample size of 1800 (2000*0.90, 600 per group) for T2 measures and a sample size of 1400 (2000*0.70, 466 per group) for T3 measures.

Elements of the primary aim outcomes (aims 1 and 2) are summarized in Table 2. For the aim 1 primary outcome (anxiety), we will compare the percent change in anxiety (STAI) from baseline between the three study groups at 1 month (T2) and 1 year (T3) following notification. We conservatively assume a standard deviation of 50 percentage points (range/4) in the percent change measure for 80% power (along with 1.7% type-I error rate) to detect a difference of 9.3 in average percent change between any two of the groups on two-sample *t* test at T2 with 600 per group. At T3, we will have 80% power to detect a difference of 10.6 (466 per group).

**Table 2:** Elements of primary outcomes

Domain	Specific measurement	Specific metric	Method of aggregation	Time point
Anxiety (aim 1)	State anxiety subscale of the State-Trait Anxiety Inventory (STAI)	Percent change in STAI score from T1 to T2, and also from T1 to T3	The distribution of the percent change in STAI will be summarized by the mean and standard deviation	Comparisons of percent change from T1 between the groups will be made at T2 and T3
MBD knowledge (aim 1)	3 survey items assessing knowledge of MBD as a risk factor, MBD as a masking effect, and mammography screening intervals	Knowledge score will be calculated as the number of the three knowledge items that are answered correctly (possible range 0–3)	The distribution of the knowledge score will be summarized with frequencies and percentages (i.e., *N* (%) of participants with all 3 items correct, *N* (%) with 2 items correct, *N* (%) with 1 item correct, and *N* (%) with no items correct)	Comparisons of knowledge score between the groups will be performed at T2 and T3
Continued adherence to mammography (aim 2)	For each participant, we will assess whether they were adherent to their next annual mammogram. This information will be captured via self-report in the T3 survey, corroborated with the electronic health record (EHR) at T3, and assessed via EHR again at T4	Participants will be classified as adherent if there is evidence of having had the next annual mammogram, and will otherwise be classified as non-adherent	Frequency and percentage of participants who were adherent	Comparisons of adherence between the groups will be performed at T3 and T4

T1 = enrollment; T2 = 2–4 weeks post-randomization; T3 = 1-year post-randomization; T4 = 2-years post-randomization

Knowledge of breast density will be measured ordinally as the number of correct items (range 0–3 correct). This will be tested using the pairwise Wilcoxon rank-sum test, for which the null hypothesis is that the probability of someone answering more items correctly in one group versus another is 0.5 (i.e., equal chance). At T2, with 600 women per group, we will have 80% power (along with 1.7% type-I error rate) to detect a probability of 0.554 or more between any two of the groups. At T3, we will have 80% power to detect a probability of 0.561 (466 per group). For anxiety and knowledge of MBD, a complete-case analysis will be conducted (excluding missing observations), and we will use the last-value-carried-forward approach for comparison.

Aim 2 will compare adherence to next routine screening mammogram between the three notification groups. This outcome “adherence to next annual screening” is pertinent to all 2000 women and will be obtained from the EHR. A sample size of 666 in each group will yield 80% power to detect a difference of 9 percentage points between any two of the groups (type-I error rate 1.7%) based on the chi-square test. Participants with no evidence of a subsequent mammogram will be treated as non-adherent to the next annual screening.

Aim 3 will qualitatively analyze documentation provided by the *promotora* after each patient interaction in the interpersonal group. Based on the recommendation of Morse [44], we will have ample data from over 600 patient-*promotora* interactions to capture a range of responses. Furthermore, this large sample size will offer the opportunity to qualitatively examine the type of information exchanged by particular subgroups of patients, such as Spanish-speaking only, those receiving notification that they have dense breasts, and those receiving notification that they do not have dense breasts. The analysis of aim 3 qualitative data will provide a rich resource for informing the design of targeted educational materials for specific populations and developing generalizable tools to enhance communication about appropriate risk-based screening for breast cancer.

### Compensation and anticipated attrition

Compensation is important for recruiting and retaining research participants [45, 46]. Women are offered remuneration equaling US$25 at completion of the T1, T2, and T3 surveys. Maximum compensation to study participants is US$75, which is unlikely to be coercive in any model of reimbursement [47]. Careful tracking procedures are used to minimize attrition including confirmation of phone numbers and addresses at follow-up study contacts. Non-response to any assessment will not preclude subsequent data collection, including subsequent survey assessments and data abstraction from the EHR at T3 and T4. If at any point a woman withdraws from the study, she will not receive future contacts and the study team will not collect additional data.

### Data monitoring, quality, and fidelity

Data monitoring is overseen by the Principal Investigator and the steering committee. The study statistician provides the Principal Investigator and steering committee with monthly reports showing allocation of participants across study groups and ranges for data values on MBD and Breast Imaging and Reporting Data System (BI-RADS) categories, age at consent, ethnicity, language preference, and body mass index (BMI). The steering committee reviews these to identify any potential issues in allocation. No interim analyses on study outcomes will be completed until the end of study recruitment.

Surveys are double data-entered by trained data management personnel to ensure data quality, and checked for consistency (i.e., skip pattern inconsistency, missing data). The study statistician completes regular review of the data to identify potential data entry or survey completion errors, and she works with the Principal Investigator and study staff as needed to develop and systematically apply data quality rules to the data set. Monthly steering committee review and review of survey data entry serve as audit and quality checks. The Sponsor receives annual progress reports but does not participate in on-going audit activities.

For the interpersonal group, the *promotora* audio records a random 20% sample of calls (with participant permission), and fidelity of delivery is evaluated against a checklist by a bilingual member of the study staff not involved in intervention delivery. The expected performance level is at least 95%. Retraining will take place if performance is below 95%.

### Data handling and protection against risk

Each participant is assigned a study number, which will be used to identify research data. Signed consent documents and research authorization forms are kept separately from the other research data to further protect participants’ confidentiality. Study data are managed using SAS version 9.4 (SAS Institute Inc., Cary, NC, USA), REDCap, and Medidata Rave® databases designed specifically for the study. These systems are located behind the institutional firewall and are accessible only to authorized personnel. The tools are HIPAA-compliant and are set up to use branching logic and field validation to help improve the accuracy of data entry. Paper surveys are kept in secure file cabinets. Audio recordings are stored on secure file servers. The steering committee and the IRB considered the educational interventions to have minimal risk, and as such no separate data safety monitoring board was required. The study team monitors withdrawals from the study, and a behavioral health plan is in place for participants whose responses to the PROMIS questions indicate potentially elevated depressed mood. Due to the educational nature of the interventions, there are no expected risks to participants that would result in termination of the trial or in discontinuation of interventions for a given trial participant. The steering committee reviews any deviations from study protocol (which are also reported to the IRB) at their monthly meeting along with data on withdrawals and participants with elevated PROMIS scores. Under the behavioral health plan, a member of the trial steering committee will contact any participants meeting a predetermined threshold on the PROMIS questions and ask whether the participant would like to be connected to a behavioral health professional at MPHC.

## Discussion

Recent years saw an uptick in the number of states adopting MBD notification legislation, and recent federal legislation further demonstrates the perceived importance of providing women with information about MBD. However, most notification letters provide information about density results without providing education on the implications of MBD. Reliance on written notification also raises important unexplored issues related to the impact of notification on women with low health literacy, language barriers, and potentially lesser access to the types of supplemental screening options most appropriate for women with dense breast tissue. Recent qualitative studies in Hispanic/Latina women suggest current notification strategies may result in confusion and worry, and misinterpretation of key implications like masking and breast cancer risk [9, 10].

Even as a federal notification mandate goes into effect, there remains a paucity of evidence on the effects of notification on screening behaviors or psychological factors, including those that potentially serve to moderate outcomes like diagnostic delay among Latinas. This study addresses these important questions in an RCT, which includes an interpersonal intervention shown to be effective in related studies of breast health conducted with populations facing similar health disparities and barriers to health. The results of this study will be widely shared via publication and presentation for the purpose of informing both healthcare delivery and public policymaking. This study will provide evidence on the effects of notification combined with educational information on behavioral and psychosocial outcomes. Understanding knowledge gaps will also inform future patient education efforts. Importantly, it will assess the relative benefits of additional written educational information alongside interpersonal education from a bilingual health educator. While additional written information may increase knowledge of MBD, this study design will provide evidence of whether interpersonal education—which allows opportunities for correcting misinformation—can further alleviate confusion and anxiety. Furthermore, documentation of patient questions can inform future educational interventions and refinement of intervention implementation, e.g., factors related to delivery of a *promotora*-led intervention. The cost analysis will likewise provide information for health systems that are seeking to adopt MBD educational strategies. Finally, this RCT will determine the impact of educational strategies on screening adherence, which is the ultimate strategy for reducing diagnostic delay and disparities in cancer mortality among Latinas.

The study is being conducted in an FQHC in Arizona that serves a high proportion of Latinas. While the study is only being conducted in a single setting, such a design leverages the potential to study outcomes while controlling for the potential of confounding with a study that enlisted multiple clinics with potentially different EHRs or spanned states with different density legislation. Limiting this study to a single site also leverages our potential to study the delivery of the intervention and to identify issues related to future sustainability and generalizability. Limitations of qualitative data collection by the *promotora* in aim 3 are minimized by the use of a theory-informed educational script and fidelity checks.

Finally, we recognize that a formal cost analysis, such as return on investment, and including detailed study of higher-resource time saved (that of a healthcare provider) due to the *promotora* intervention would provide a more refined ability to compare costs across the study groups, however, these organizational-level analyses are beyond the scope of this study and potentially not generalizable. The approach we have adopted instead will provide important cost data to deliver the educational interventions in any clinic setting, should they demonstrate beneficial outcomes.

## Trial status

Ongoing. Recruitment and randomization of participants began on 27 October 2016. The current trial protocol is version 5 (approved 22 May 2019). Recruitment is expected to be complete 30 November 2019.

## Supplementary information

**Additional file 1.** SPIRIT 2013 Checklist: Recommended items to address in a clinical trial protocol and related documents.

## Data Availability

The full protocol, datasets used and/or analyzed during the current study, and statistical code, may be made available from the corresponding author on reasonable request after one year from the final publication on the primary aims from the corresponding author and with appropriate resources.

## Abbreviations

EHR: Electronic health record
FQHC: Federally qualified health center
HIPAA: Health Insurance Portability and Accountability Act
LLEAD: Latinas LEarning About Density
MBD: Mammographic breast density
MPHC: Mountain Park Health Center
PROMIS: Patient-Reported Outcomes Measurement Information System
PROs: Patient-reported outcomes
RCT: Randomized clinical trial
STAI: State-Trait Anxiety Inventory
